# Characteristics Associated With High-Intensity Binge Drinking in Alcohol Use Disorder

**DOI:** 10.3389/fpsyg.2021.750395

**Published:** 2021-10-20

**Authors:** Joshua L. Gowin, Matthew E. Sloan, James K. Morris, Melanie L. Schwandt, Nancy Diazgranados, Vijay A. Ramchandani

**Affiliations:** ^1^Department of Radiology, University of Colorado Anschutz Medical Campus, Aurora, CO, United States; ^2^Laboratory on Human Psychopharmacology, National Institute on Alcohol Abuse and Alcoholism, Bethesda, MD, United States; ^3^Addictions Division, Centre for Addiction and Mental Health, Toronto, ON, Canada; ^4^Centre for Addiction and Mental Health, Campbell Family Mental Health Research Institute, Toronto, ON, Canada; ^5^Division of Neurosciences and Clinical Translation, Department of Psychiatry, University of Toronto, Toronto, ON, Canada; ^6^Department of Pharmacology and Toxicology, University of Toronto, Toronto, ON, Canada; ^7^Office of the Clinical Director, National Institute on Alcohol Abuse and Alcoholism, Bethesda, MD, United States

**Keywords:** binge alcohol consumption, elastic net, random forests, substance use and misuse, alcohol

## Abstract

High-intensity binge drinking, defined as consuming 2–3 times the level of a binge (4 or 5 drinks for women or men), increases the risks of overdose and alcohol-related cancer relative to lower levels of drinking. This study examined the relationship between high-intensity binge drinking and three domains hypothesized to contribute to alcohol use disorder (AUD): incentive salience, negative emotionality, and executive function. This cross-sectional study at the National Institute on Alcohol Abuse and Alcoholism examined 429 adults with AUD and 413 adults without a history of AUD. Drinking was assessed using the 90-day Timeline Followback interview. The AUD sample was divided into training and testing sets, and a machine learning model was generated in the training set and then applied to the testing set, to classify individuals based on if they had engaged in high-intensity binge drinking. We also conducted regression models for the following dependent variables: the presence of high-intensity binge drinking, frequency of high-intensity binge drinking, and number of drinks per of binge. Independent variables in these regression models were determined by variable selection from the machine learning algorithm and included time thinking about alcohol, depression rating, and positive urgency as representative variables for the three domains. These variables were assessed using self-report measures. The models were applied to the adults without a history of AUD to determine generalizability. The machine learning algorithm displayed reasonable accuracy when classifying individuals as high-intensity binge drinkers (area under ROC=0.74, 95% CI 0.67, 0.80). In adults with AUD, greater depression rating (OR=1.04, 95% CI 1.01, 1.070) and amount of time thinking about alcohol (OR=1.48, 95% CI 1.20, 1.91) were associated with greater likelihood of high-intensity binge drinking. They were also associated with greater frequency of high-intensity binge drinking days and greater number of drinks on binge occasions. Our findings suggest that incentive salience may contribute to high-intensity binge drinking in both controls and individuals with AUD. Negative emotionality was only associated with high-intensity binge drinking in individuals diagnosed with AUD, suggesting that it may be a consequence rather than a cause of high-intensity binge drinking.

## Introduction

Alcohol use disorder (AUD) affects many lives each year and contributes to thousands of premature deaths (Rehm et al., 2009, 2015). AUD indicates that an individual has impaired ability to control alcohol use despite negative consequences, but the actual patterns of alcohol misuse can vary substantially. One type of alcohol misuse, binge drinking, is defined as achieving a blood alcohol concentration above 0.08g/dl, which requires consuming approximately 4 drinks for a typical female or 5 drinks for a typical male in a two-hour period (National Institute on Alcohol Abuse and Alcoholism, 2004). Some individuals engage in high-intensity binging where they drink 2–3 times that amount (Patrick et al., 2016, 2021; Hingson et al., 2017). Binge drinking episodes increase the risk of traumatic injuries and suicidal behavior (Schaffer et al., 2008), and chronic binge drinking increases the likelihood of developing cancer and ischemic heart disease (Ruidavets et al., 2010; Griswold et al., 2018). Epidemiological studies of young adults have shown that high-intensity binging is more common in rural relative to urban areas, among users of nicotine or cannabis (Patrick et al., 2013), and in individuals with depressive symptoms (Patrick et al., 2021). The literature that characterizes individuals who exhibit high-intensity binge drinking comes largely from epidemiological studies, but few studies have examined high-intensity binge drinking in adults with AUD. It therefore remains unclear which psychological factors are associated with high-intensity binge drinking in clinical samples.

According to leading theories of AUD etiology, three domains contribute to uncontrolled drinking and preoccupation with alcohol (Koob and Volkow, 2010; Kwako et al., 2016). First, incentive salience describes high levels of desire and focus on obtaining alcohol. Second, negative emotionality indicates the degree of symptoms like withdrawal, anxiety, or irritability present at a trait-level or as a state-level consequence of not consuming alcohol. Third, executive function describes ability to consider long-term consequences of choices and make decisions that support the individual’s goals. Recent analysis of a group of adults representing a broad spectrum of drinking, from light to heavy, confirmed that their symptoms and phenotypes could be mapped onto those three domains (Kwako et al., 2019b). Adults with AUD are more likely to have a mood disorder such as depression relative to adults without alcohol use disorder (Grant et al., 2015). Adults with AUD also demonstrate poorer executive function, such as problem solving, inhibitory control (Stephan et al., 2017), and delay discounting (Gowin et al., 2019). Craving alcohol, a metric of incentive salience, has also been associated with a greater risk of AUD (Keyes et al., 2011). Adults with AUD, relative to a comparison group, showed greater activation of reward-related brain areas, such as the nucleus accumbens when viewing alcohol pictures (Sjoerds et al., 2014), suggesting that enhanced incentive salience relates to greater neural reward signaling in response to alcohol cues. Likelihood of reaching binge-level exposure in laboratory models of alcohol consumption is higher among individuals with lower levels of executive control (Gowin et al., 2017). While some studies have examined pieces of this model, few have examined these three factors collectively as indicators of high-intensity binge drinking in adults with AUD.

To examine whether the domains of executive function, incentive salience, and negative emotionality were associated with high-intensity binging, we used data collected from a large sample of adults with AUD. In addition to looking at the three domains, we also looked at clinical and demographic factors including family history of AUD and comorbid substance use disorder diagnoses. We used a machine learning approach to examine many variables concurrently and to reduce systematically the number of variables to generate parsimonious models. Lastly, to determine generalizability, we applied the models to data from adults without a history of AUD.

## Materials and Methods

### Participants

Participants were recruited at the National Institutes of Health (NIH) Clinical Center in Bethesda, Maryland for protocols that aim to characterize individuals by collecting a battery of measures over the course of one or 2days. Some participants were seeking treatment for alcohol misuse, and others were seeking to participate in research without treatment. The NIH intramural institutional review board approved these protocols. All participants provided consent prior to data collection. Individuals who were pregnant, less than 18years of age, or who were unable to provide consent were excluded. We identified 1,140 individuals with a current alcohol use disorder. Many of these individuals completed only a subset of measures, so a final sample of 429 adults with complete data for the negative emotionality, executive function, and incentive salience measures was included in this study. We also identified 721 adults with no history of alcohol use disorder and retained a final sample of 413 adults with complete data. This study was entered into the Open Science Framework registry (doi: 10.17605/OSF.IO/ZMQVH).

### Drinking Outcomes

Participants completed a 90-day Timeline Followback interview (Sobell et al., 1996) to assess alcohol consumption on each of the 90days prior to enrollment. The primary outcome was a binary classification of having engaged in any level III high-intensity binge drinking occasions. High-intensity binging was defined as any instance of consuming at least three times the binge level (i.e., 12+ drinks in a day for females or 15+ drinks in a day for males). This allowed for relatively balanced groups since only 73 individuals (17.0% of the AUD sample) never engaged in level II high-intensity binge drinking (2×binge), but 178 individuals (41.5% of the AUD sample) never engaged in level III high-intensity binge drinking (3x binge). We also examined frequency of high-intensity binge drinking across the 90-day assessment period and intensity of binge drinking by determining the average number of drinks on days where the participant consumed >4 or>5 drinks for a female or male, respectively. For adults with no history of AUD, few individuals engaged in high-intensity binge drinking (*N*=15) and high-intensity drinking was infrequent (Median=2days out of 90). As a result, we limited our analyses to examining predictors of whether or not an individual had engaged in any level II high-intensity binge drinking occasions and examining predictors of the number of drinks consumed per binge drinking occasion.

### Procedures

Participants completed a Structured Clinical Interview for DSM-IV (First et al., 2002) or DSM-5 (First et al., 2015) to assess for psychiatric disorders. All participants included in the primary analysis had a current diagnosis of alcohol abuse, dependence, or alcohol use disorder, depending on if they were assessed by SCID-IV or SCID-5. Participants included in the non-AUD group had no current or past diagnoses of an alcohol use disorder. We additionally looked at the presence of comorbid substance use disorders including cannabis use disorder, stimulant use disorder, opioid use disorder, inhalant use disorder, sedative use disorder, and PCP use disorder (or a substance abuse or dependence diagnosis for those assessed with DSM-IV). The Alcohol Use Disorder Identification Test (Saunders et al., 1993) and Alcohol Dependence Scale (Skinner and Horn, 1984) were collected to determine severity of alcohol use problems.

### Negative Emotionality

Depression, anxiety, neuroticism, and aggression were included as the hypothesized measures of negative emotionality, consistent with a factor analysis (Kwako et al., 2019a). Participants completed the Montgomery-Asberg Depression Rating Scale (Svanborg and Asberg, 1994) as part of the Comprehensive Psychopathological Rating Scale (Perris et al., 1978) to assess depressive symptoms and the State Trait Anxiety Inventory (Spielberger, 2010) to assess anxiety symptoms. The Buss Perry Aggression Questionnaire (Buss and Perry, 1992) was used to assess aggressive behavior, which although a behavior rather than an emotion, often stems from anger and negative emotions (Berkowitz, 1998). The neuroticism scale of the NEO Personality Inventory (Costa and McCrae, 2008) was also included as a hypothesized negative emotionality factor.

### Executive Function

Conscientiousness, delay discounting, and impulsivity were the hypothesized measures of executive function. Conscientiousness was assessed using the NEO Personality Inventory (Costa and McCrae, 2008). To assess delay discounting, participants completed a 66-item monetary choice task (Gowin et al., 2019). For each choice, participants chose between an option to receive $100 after a delay of 7 to 30days or a smaller amount of money now (e.g., $90 now or $100 in 30days). The point of indifference for each delay (7, 14, 20, 25, or 30days) was defined as the amount where the participants switched between the smaller amount now to the larger amount later. The degree of discounting was defined by the delay discounting constant, k, derived from the hyperbolic discounting function developed by Mazur (1987). As the distribution of *k* is highly skewed, we used the natural log, *ln k*, for analyses. To assess impulsivity, participants completed both the Barratt Impulsiveness Scale version 11 (Patton et al., 1995) and the UPPS-P (Lynam et al., 2006).

### Incentive Salience

Items 1, 11, and 13 of the Obsessive–Compulsive Drinking Scale (Anton et al., 1995) were the measures of the incentive salience factor based on prior work from our group (Kwako et al., 2019b). The items were Likert rated from 0 to 4. Item 1 asks, “How much of your time when you are not drinking is occupied by ideas, thoughts, impulses, or images related to drinking?” Item 11 asks “If you were prevented from drinking alcohol when you desired a drink, how anxious or upset would you become?” Item 13 asks “How strong is the drive to consume alcoholic beverages?”

### Clinical, Demographic, and History Measures

We examined whether participants had a comorbid substance use disorder and scored this as a binary variable. The Fagerström Test for Nicotine Dependence (Heatherton et al., 1991) was used to determine tobacco use, and non-smokers were scored as 0. We asked each participant at what age they had their first alcoholic drink. We also collected information about years of education and household income as part of a demographic questionnaire. We assessed family history of alcohol misuse using the Family Tree Questionnaire (Vogel-Sprott et al., 1985) and included a single binary variable, family history, that indicated either the presence or absence of relatives with a history of alcohol problems. We also included age and sex.

### Machine Learning Classification Analysis

For the AUD group, we used a machine learning algorithm to determine whether we could classify individuals as having engaged with high-intensity binge drinking using the caret package^1^ in R version 3.6.3 (Kuhn, 2008). We first created two equally sized subsamples using “createDataPartition,” so that the proportion of participants who engaged in high-intensity binging was equivalent in the training and testing sample. This resulted in 89 individuals with no high-intensity binge occasions in both the training and testing samples. The training group had 126 individuals with a high-intensity binge occasion, while the testing group had 125 such individuals. Next, we selected three algorithms (elastic net, random forest, and support vector machine), that function well when there are many predictor variables but a small number of observations (i.e., large *p*, small *n*; Zou and Hastie, 2005). The outcome for the analysis was the binary variable indicating whether a participant engaged in any high-intensity binge drinking occasions. We used all the predictor variables. Using these variable sets, we conducted 10-fold cross-validation for each algorithm, repeated 20 times to optimize parameters in the algorithms. The goal of each algorithm was to maximize area under the receiver operating characteristic curve. We then used 10-fold cross-validation to generate an ensemble model comprised of a linear combination of the three optimized algorithms.

The ensemble model was applied to the testing sample to generate an estimate of the probability that an individual engaged in high-intensity binge drinking. Each individual’s probability was compared to their status as having engaged in high-intensity binge drinking using receiver operatic characteristic (ROC) analysis to determine whether the predictions performed better than chance. Significance was defined as when the lower bound of the 95% confidence interval for the area under the ROC plot exceeded 0.5 using DeLongs method. We also report the Brier score, which is equivalent to mean squared error for binary classification (Brier, 1950). Lastly, we used a cutoff of probability greater than 0.5 to generate a binary classification of high-intensity binge drinking and examined whether the accuracy was significantly greater than the no-information rate.

### Machine Learning Variable Reduction

For the AUD group, we also used a machine learning approach to reduce the number of variables to include in linear regression models. Using the entire sample of adults with an AUD, we generated variable importance scores for the four sets of independent variables: incentive salience, negative emotionality, executive function, and clinical/demographic/history measures. For random forest, the R package describes the process for determining variable importance as “for each tree, the prediction accuracy on the out-of-bag portion of the data is recorded. Then, the same is done after permuting each predictor variable. The difference between the two accuracies is then averaged over all trees and normalized by the standard error.” For elastic net, the absolute value of the t-statistic for each model parameter is used. For support vector machine, the algorithm computes the area under the ROC curve. For all models, values are scaled so that the sum of all variable importance scores equals 100. For the ensemble, variable importance is calculated as an average of the product of the variable importance from each variable in a given model by the weight of that model in the ensemble. For a variable to be selected, it needed to have an ensemble variable importance score greater than 10. To reduce redundancy and the possibility of collinearity, we examined the correlation between potential variables and removed the variable with a lower importance score if two variables had a correlation greater than *r*=0.35.

### Analytic Approach

To compare group characteristics, we tested for group differences of binary variables using chi-squared tests. To determine group differences for continuous measures, we used t tests if the variables were normally distributed and Mann-Whitney U tests if the variables were not normally distributed. Due to the number of tests, we used a Benjamini-Hochberg false discovery rate correction so that an uncorrected *p*<0.009 corresponded to a corrected *p*<0.05. We computed effect sizes using Cohen’s *d,* where a value >0.29 corresponded to a corrected *p*<0.05.

For the AUD group, using the most important variables from each domain, we conducted analyses for three outcomes, including status as having engaged in any high-intensity binge occasions, the number of high-intensity binge occasions out of 90, and the average number of drinks per binge drinking occasion. We used binary logistic regression to examine status as having engaged in high-intensity binge drinking. Among only the individuals who reported at least one high-intensity binge occasion (*N*=251), we used multiple linear regression to examine predictors of frequency of high-intensity binge occasions. Among only individuals who reported at least one binge occasion (*N*=424), we used multiple linear regression to examine predictors of average drinks per binge occasion. We included treatment seeking status in each model since we have previously observed demographic differences with respect to treatment and non-treatment seekers (Rohn et al., 2017). We used a two-tailed test of *p*<0.05 to determine significance.

We conducted two additional analyses to assess whether our findings in the AUD sample would extend to a sample of individuals without AUD. First, we employed binary logistic regression to examine predictors of whether an individual had ever engaged in a high-intensity binge drinking episode using the same set of predictors as the AUD analysis. Second, we used linear regression to examine predictors of average drinks per binge drinking occasion in individuals who had reported at least one binge drinking occasion (*N*=127).

## Results

### Group Characteristics in the Sample With a Current AUD Diagnosis

**Figure 1:**
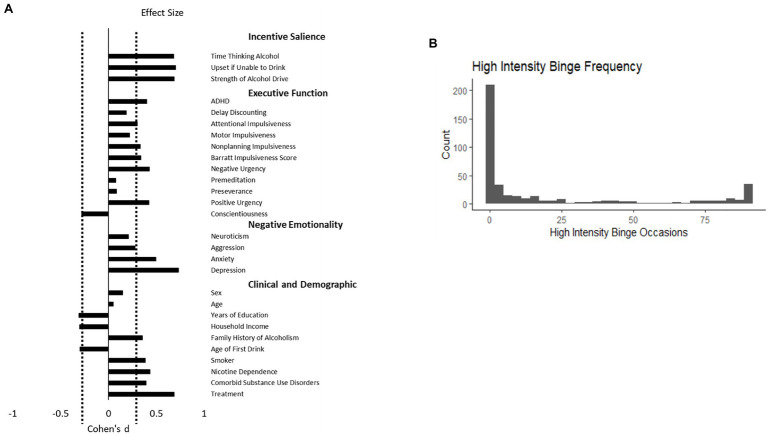
(A) depicts the effect size of the group difference between adults with alcohol use disorder who engaged in high-intensity binge drinking behavior relative to those who did not. Attentional, Motor, and Nonplanning Impulsiveness are subscales of the Barratt Impulsiveness Scale. Delay discounting is the natural log of the *k* discounting constant. The three incentive salience measures represent questions 1, 11, and 13 from the Obsessive–Compulsive Dependence Scale. Positive values indicate that the group that engaged in high-intensity binge drinking had greater values of the measure. The dashed line represents a significant difference at a false discovery rate corrected *value of p* <0.05. (B) depicts the frequency of each value high-intensity binge occasions across 90days in this sample of 429 adults with alcohol use disorder.

Adults with AUD who engaged in high-intensity binge were compared to those who did not engage in high-intensity binge drinking (Table 1). Individuals who engaged in high-intensity binge drinking were more likely to smoke, have a comorbid substance use disorder, display higher levels of nicotine dependence severity, and have a lower level of education (corrected values of *p* <0.05 for all comparisons). Individuals who engaged in high-intensity binge drinking also scored higher on the Obsessive–Compulsive Drinking Scale, demonstrated greater positive urgency, and had higher scores for depression and aggression (corrected values of *p* <0.05 for all comparisons). The largest group differences were observed for incentive salience variables, depression rating, and whether the participant was seeking treatment (Figure 1).

**Table 1:** Group characteristics.

	AUD with High-Intensity Binge (*N*=251)		AUD without High-Intensity Binge (*N*=178)		Chi-square – value
	*N*	%	*N*	%	
Male	186	74.1	120	67.4	2.0
Smoker	149	59.4	72	40.4	14.2^*^
Comorbid substance use disorders^a^	157	62.5	77	43.3	14.9^*^
Treatment seeker	159	63.3	55	30.9	42.6^*^
	**Mean**	**SD**	**Mean**	**SD**	**t-value**
Age (years)	43.5	12.5	42.9	12.9	−0.4
AUDIT score	26.2	7.9	17.9	8.1	−10.6^*^
Alcohol Dependence Scale Score	19.9	8.8	11.6	7.8	−9.4^*^
Delay Discounting ln (k)	−3.7	1.6	−3.9	1.4	−1.7
Depression rating^c^	13.0	10.2	6.5	7.6	−7.6^*^
Anxiety rating^d^	45.0	12.8	38.9	12.0	−5.1^*^
Aggression score^e^	70.5	20.7	65.1	18.2	−2.8^*^
Impulsivity score^f^	67.3	12.6	63.1	12.0	−3.5^*^
Positive Urgency	2.2	0.7	1.9	0.7	−4.4^*^
OCDS Q1	1.9	1.2	1.2	0.9	−7.1^*^
OCDS Q11	1.8	1.2	1.1	0.9	−7.3^*^
OCDS Q13	2.2	1.2	1.4	1.0	−7.1^*^
	**Median**	**IQR**	**Median**	**IQR**	**U-value**
Nicotine dependence severity^g^	1.0	5.0	0.0	2.8	17,377^*^
Binge drinking days^b^	79.0	39.0	34.0	48.8	10,924^*^
High-intensity binge drinking days^b^	23.0	72.0	0.0	0.0	–
Average drinks per binge^b^	14.3	8.1	6.5	2.3	3,587^*^
Total drinks^b^	986.0	929.0	322.5	299.3	6,365^*^

a Diagnosis of cannabis or stimulant use disorder or abuse or dependence if assessed with SCID-IV.

b Assessed by the 90-day timeline followback.

c Assessed by the Montgomery-Asberg Depression Rating Scale.

d Assessed by the Spielberger State Trait Anxiety Inventory.

e Assessed by the Buss Perry Aggression Questionnaire.

f Assessed by the Barratt Impulsivity Scale version 11.

g Assessed by the Fagerstrom Test for Nicotine Dependence.

* denotes *p*<0.05 with Benjamini-Hochberg correction.

Individuals who engaged in high-intensity binge drinking had greater alcohol dependence severity as measured by the AUDIT and Alcohol Dependence Scale (corrected *p*<0.05). They also reported more binge drinking days out of the past 90 on the Timeline Followback and more total drinks (corrected *p*<0.05). On average, individuals in the high-intensity binge drinking group tended to exceed the threshold for an high-intensity binge once every 4days. The distribution of high-intensity binge drinking occasions is depicted in Figure 1, Panel B.

### Group Characteristics in the Sample With No History of AUD

The individuals who had engaged in high-intensity binge drinking were younger and had higher AUDIT scores than individuals with no high-intensity binge occasions (*p*<0.05, Table 2). They also had higher values for the Obsessive–Compulsive Dependence Scale measures (*p*<0.05). The groups did not differ on other demographic or domain metrics (Table 2).

**Table 2:** Group characteristics.

	Never AUD with High-Intensity Binge (*N*=15)		Never AUD without High-Intensity Binge (*N*=398)		Chi-square – value
	*N*	%	*N*	%	
Male	9	0.6	186	0.5	0.56
Smoker	2	0.1	19	0.1	–
Comorbid substance use disorders^a^	0	0.0	18	0.1	–
	**Mean**	**SD**	**Mean**	**SD**	**t-value**
Age (years)	28.99	9.37	37.26	13.43	3.29^*^
AUDIT score	8.87	4.16	2.59	2.37	−5.82^*^
Delay Discounting ln (k)	−4.23	1.42	−4.49	1.57	−0.611
Depression rating^c^	1.80	2.91	1.10	2.61	−0.918
Anxiety rating^d^	31.07	6.94	28.08	6.85	−1.64
Aggression score^e^	54.53	11.87	51.83	13.97	−0.859
Impulsivity score^f^	55.87	9.63	51.70	8.13	−1.654
Positive Urgency	1.54	0.49	1.36	0.44	−1.367
	**Median**	**IQR**	**Median**	**IQR**	**U-value**
OCDS Q1	0.0	1.0	0.00	0.00	2182.5^*^
OCDS Q11	0.0	1.0	0.00	0.00	2121.5^*^
OCDS Q13	0.0	1.0	0.00	0.00	1881^*^
Nicotine dependence severity^g^	0.0	0.0	0.00	0.00	2,562.50
Binge drinking days^b^	19.0	20.5	0.00	1.00	218^*^
High-intensity binge drinking days^b^	2.0	9.5	0.00	0.00	–
Average drinks per binge^b^	8.4	3.5	5.00	1.51	186^*^
Total drinks^b^	178.0	187.3	15.00	38.75	400.5^*^

a Diagnosis of cannabis or stimulant use disorder or abuse or dependence if assessed with SCID-IV.

b Assessed by the 90-day timeline followback.

c Assessed by the Montgomery-Asberg Depression Rating Scale.

d Assessed by the Spielberger State Trait Anxiety Inventory.

e Assessed by the Buss Perry Aggression Questionnaire.

f Assessed by the Barratt Impulsivity Scale version 11.

g Assessed by the Fagerstrom Test for Nicotine Dependence.

* denotes *p*<0.05 with Benjamini-Hochberg correction.

### Machine Learning Classification Analysis

**Figure 2:**
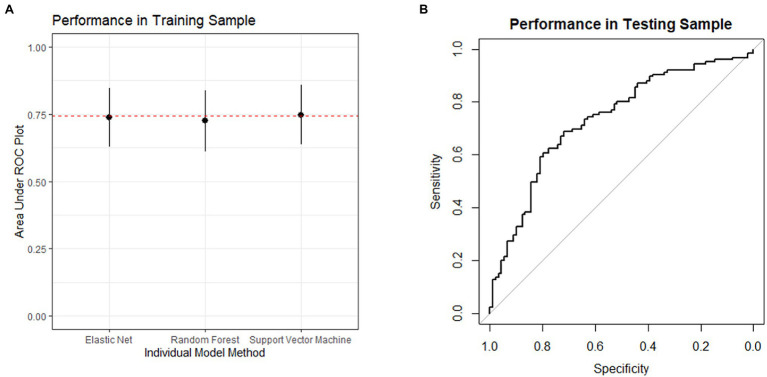
(A) depicts the performance in the training sample during model development. The performance of each model in the algorithm is depicted as the mean area under the receiver operating characteristic plot. The error bars represent the standard deviation. The dashed line represents the performance of the ensemble model. (B) depicts the receiver operating characteristic plot for the model’s performance when applied to the testing sample. The area under the curve is 0.74, and the lower bound of the 95% confidence interval is 0.67.

In the training set, the ensemble model had an area under the ROC of 0.74, with a sensitivity of 0.55 and a specificity of 0.79, indicating reasonable model performance. We applied this model to the testing set and observed similar performance, with area under the ROC of 0.74 (95% confidence interval 0.67–0.80) and a Brier score of 0.20, indicating significant improvement over chance (Figure 2). Using a probability of greater than 0.5 as a cutoff for classification, the accuracy of the model was 0.68 [95% CI 0.61–0.74, *p*=0.003 (accuracy > no-information rate)]. The sensitivity was 0.54 and the specificity was 0.78.

### Machine Learning Variable Reduction

For incentive salience, all three questions from the Obsessive–Compulsive Dependence Scale had importance scores above 10, with question 1 as the highest (51.6), followed by question 11 (33.4), and question 13 (15.0). Question 1 was correlated with each of the other variables with a Pearson’s coefficient *r*>0.35, so it was the only variable included in the final model. For negative emotionality, depression rating (74.9) was the only variable with an importance score above 10. For executive function, positive urgency (60.2) was the only variable with importance score above 10. For the clinical, demographic, and history variables, the presence of comorbid substance use disorders (26.0), family history of alcohol misuse (15.4), sex (11.6), age of first drink (11.3), and household income (11.0) met the criteria and their correlation coefficients were less than 0.35, so all were included.

### Markers Associated With Presence of High-Intensity Binge Drinking

**Figure 3:**
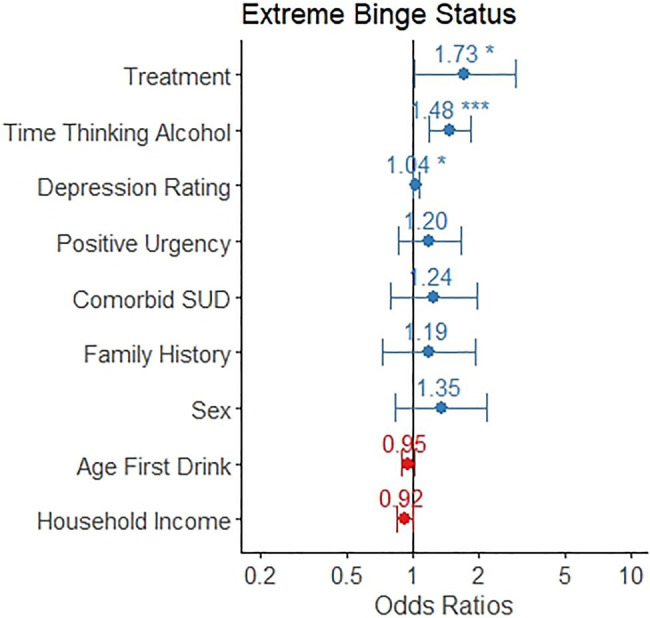
The odds ratio for each variable as it relates to likelihood of any engagement of high-intensity binge drinking is depicted, along with a 95% confidence interval. Individuals in treatment, those who spent more time thinking about alcohol, and those with greater depression ratings were more likely to engage in high-intensity binge drinking.

A binary logistic regression model for the presence of high-intensity drinking occasions found that treatment seeking status (odds ratio=1.73, 95% CI: 1.01, 2.95), greater time spent thinking about alcohol (odds ratio=1.48, 95% CI: 1.20, 1.85), and higher scores for depression rating (odds ratio=1.04, 95%CI: 1.01, 1.07) significantly predicted high-intensity binge drinking (Table 3 and Figure 3). Positive urgency, the presence of a comorbid substance use disorder, age of first drink, sex, family history of alcohol misuse, and household income were not significant predictors of high-intensity binge drinking.

**Table 3:** Reduced variable logistic regression.

	Odds Ratio	95% CI
Time thinking about alcohol^1^	1.48^*^	1.20, 1.85
Depression rating^2^	1.04^*^	1.01, 1.07
Positive urgency	1.20	0.87, 1.66
Treatment seeking^3^	1.73^*^	1.01, 2.95
Comorbid substance use disorder^4^	1.26	0.79, 1.98
Male sex^5^	1.36	0.84, 2.22
Family history of alcohol misuse^6^	1.20	0.73, 1.96
Age of first drink	0.96	0.90, 1.02
Household income	0.92	0.85, 1.00

1 Obsessive–Compulsive Drinking Scale question 1, scored 0–4.

2 Score from the Montgomery-Asberg Depression Rating Scale.

3 Reference group is non-treatment seeking.

4 Reference group is absence of comorbid substance use disorder.

5 Reference group is female sex.

6 Reference group is the absence of family history of alcohol misuse.

* Denotes *p*<0.05.

### Markers Associated With High-Intensity Binge Drinking Frequency

**Figure 4:**
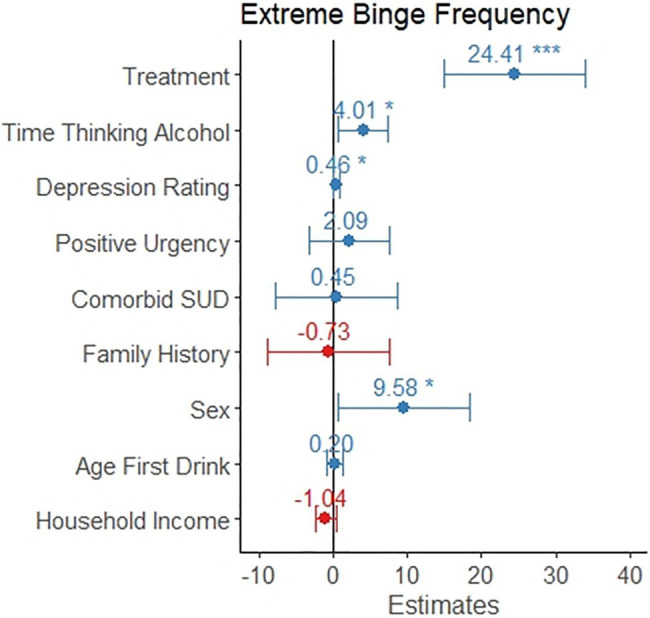
The coefficient is depicted for each variable as it relates to frequency of high-intensity binge drinking. This model only included the 251 individuals reporting at least one high-intensity binge drinking occasion. The error bars represent the 95% confidence interval. Individuals in treatment reported 24 more high-intensity binge drinking occasions. Reporting a one-point increase in time thinking about drinking was associated with four additional high-intensity binge occasions. Scoring two points higher on the Montgomery-Asberg Depression Rating Scale was associated with one additional high-intensity binge occasion. Males reported almost 10 additional high-intensity binge occasions, on average.

A linear regression model for number of high-intensity binge drinking occasions out of the prior 90days was significant (*F*_9,241_=10.0, *p*<0.001) and explained 24.5% of the variance in number of high-intensity binge occasions. Seeking treatment, spending more time thinking about alcohol, reporting greater depression rating, and being male were associated with a greater number of high-intensity binge drinking occasions (*p*<0.05, see Table 4 and Figure 4). Positive urgency, having a comorbid substance use disorder, having a family history of alcohol misuse, age of first drink, and household income were not significant predictors of number of high-intensity binge drinking occasions.

**Table 4:** Number of high-intensity binge occasions linear model.

	Coefficient	95% CI	t-value
Time thinking about alcohol^1^	4.01	0.59, 7.43	2.31^*^
Depression rating^2^	0.46	0.00, 0.93	1.98^*^
Positive urgency	2.10	−3.34, 7.54	0.76
Treatment seeking^3^	24.41	14.90, 33.93	5.06^***^
Comorbid substance use disorder^4^	0.45	−7.85, 8.75	0.11
Male sex^5^	9.58	0.67, 18.50	2.12^*^
Family history of alcohol misuse^6^	−0.73	−8.93, 7.47	−0.18
Age of first drink	0.20	−0.82, 1.23	0.39
Household income	−1.04	−2.46, 0.39	−1.43

1 Obsessive–Compulsive Drinking Scale question 1, scored 0–4.

2 Score from the Montgomery-Asberg Depression Rating Scale.

3 Reference group is non-treatment seeking.

4 Reference group is absence of comorbid substance use disorder.

5 Reference group is female sex.

6 Reference group is the absence of family history of alcohol misuse.

* Denotes *p*<0.05.

*** Denotes *p*<0.001.

### Factors Associated With Binge Drinking Intensity

**Figure 5:**
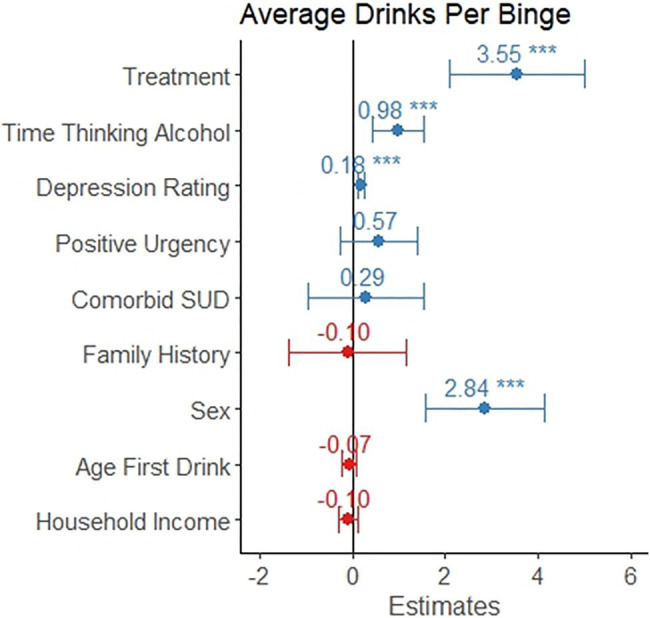
The coefficient is depicted for each variable as it relates to intensity of binge drinking, which is measured as average number of drinks on days where the participant consumed above the binge level (4+/5+ for females/males, respectively). This model only included the 424 individuals reporting at least one binge drinking occasion. The error bars represent the 95% confidence interval. Individuals in treatment reported 3.5 more drinks per binge. Reporting a one-point increase in time thinking about drinking was associated with and additional drink per binge occasion. Scoring five points higher on the Montgomery-Asberg Depression Rating Scale was associated with one additional drink per binge occasion. Males reported almost 3 additional drinks per binge occasion, on average.

A linear regression model was significant (*F*_9,414_=23.9, *p*<0.001) and explained 32.8% of the variance in number of drinks per binge occasions. Seeking treatment, spending more time thinking about alcohol, reporting greater depression rating, and being male were associated with a greater number of drinks per binge (*p*<0.05, see Table 5 and Figure 5). Positive urgency, having a comorbid substance use disorder, having a family history of alcohol misuse, age of first drink, and household income were not significant predictors of number of drinks per binge.

**Table 5:** Average drinks per binge occasion linear model.

	Coefficient	95% CI	t-value
Time thinking about alcohol^1^	0.98	0.43, 1.53	3.54^***^
Depression rating^2^	0.18	0.10, 0.26	4.58^***^
Positive urgency	0.57	−0.27, 1.41	1.33
Treatment seeking^3^	3.55	2.10, 5.01	4.80^***^
Comorbid substance use disorder^4^	0.29	−0.96, 1.54	0.45
Male sex^5^	2.84	1.56, 4.12	4.36^***^
Family history of alcohol misuse^6^	−0.10	−1.38, 1.17	−0.16
Age of first drink	−0.07	−0.22, 0.08	−0.96
Household income	−0.10	−0.31, 0.12	−0.87

1 Obsessive–Compulsive Drinking Scale question 1, scored 0–4.

2 Score from the Montgomery-Asberg Depression Rating Scale.

3 Reference group is non-treatment seeking.

4 Reference group is the absence of comorbid substance use disorder.

5 Reference group is female sex.

6 Reference group is absence of family history of alcohol misuse.

*** Denotes *p*<0.001.

### Models in the Sample Without a History of AUD

A binary logistic regression model for the presence or absence of high-intensity drinking occasions found that greater time spent thinking about alcohol (odds ratio=4.05, 95% CI: 1.43, 10.89) significantly predicted high-intensity binge drinking. None of the other variables were significant predictors of high-intensity binge drinking.

A multiple linear regression model of average drinks per binge occasion found that only sex was a significant predictor (coefficient=1.70, 95% CI 1.11, 2.28).

## Discussion

This study examined factors from a three-domain framework of alcohol use disorder to determine whether they were associated with the presence or absence of high-intensity binge drinking in a sample with AUD and with a healthy comparison group. Variables representing negative emotionality, incentive salience, and executive function included depression rating, time thinking about alcohol, and positive urgency, respectively. The results indicated that higher depression rating and time thinking about alcohol were associated with a greater likelihood of high-intensity binge drinking in adults with a current diagnosis of AUD. The results did not support an association between positive urgency and the presence high-intensity binge drinking. Furthermore, depression rating and time thinking about alcohol were associated with the frequency of high-intensity binge drinking episodes and the average number of drinks consumed per binge drinking occasion. We also found that treatment seeking status and male sex were associated with greater frequency of high-intensity binge drinking and greater intensity of binge drinking occasions. The relationship between depression rating and binge drinking did not generalize to adults without a history of AUD, but the relationship with time thinking about alcohol did. This may suggest that time thinking about alcohol contributes to alcohol misuse prior to the onset of AUD, but that higher depression rating develops as a result of prolonged high-intensity drinking and is not a causative factor, although prospective studies would be needed to address this.

Depression rating showed a strong relationship with high-intensity binge drinking. A meta-analysis suggests that the presence of either depression and alcohol use disorder doubles the risk of the other, but the risk was not due to shared etiology, and instead was due to a causal relationship (Boden and Fergusson, 2011). Structural equation modeling of longitudinal data assessing the presence of alcohol use disorder and depression between the ages of 18 and 25 suggested that the most likely pathway was that alcohol use disorder caused depression, rather than vice versa (Fergusson et al., 2009). However, the relationship may be more complex, with a positive feedback loop such that alcohol use leads to depressive symptoms, which in turn leads to further alcohol use. For example, individuals with comorbid depression and AUD are more likely to be hospitalized (Sullivan et al., 2005) and have a higher chance of relapse to alcohol use after treatment (DeVido and Weiss, 2012).

Individuals who engaged in high-intensity binge drinking had significantly higher values for each of the incentive salience measures. These variables assess time thinking about alcohol, degree of distress if denied access to alcohol, and the strength of craving. Time thinking about alcohol (question 1) was a significant predictor of high-intensity binge drinking when adjusting for other variables, and it also predicted high-intensity binge drinking frequency and binge drinking intensity. Craving has been linked to a greater likelihood of relapse following treatment (Bottlender and Soyka, 2004). Treatment with naltrexone has also been shown to diminish craving (Chick et al., 2000). Recent studies have shown that craving improves models of drinking, supporting the decision to include it as a criterion for AUD in the DSM-5 (Saha et al., 2020). Our results suggest that treatments targeting craving and alcohol salience may be expected to reduce high-intensity binge drinking, which has been confirmed in clinical trials (Martino et al., 2019). Whether targeting both craving and depression with pharmacotherapy could have additive benefits requires further study; for example, there is currently mixed evidence as to whether adding antidepressants improves alcohol outcomes in individuals with comorbid AUD and major depressive disorder (Yoon and Petrakis, 2018).

We expected to find evidence of a relationship between high-intensity binge drinking and positive urgency, age of first drink, and family history of alcohol misuse. Although these variables have been consistently linked to alcohol misuse, the lack of a relationship with high-intensity binge drinking in this sample may have several causes. First, the primary analysis examined a specific type of alcohol misuse in a sample with AUD, so there may have been a ceiling effect where the participants in this sample all had similarly high levels of positive urgency, similar family histories of alcohol misuse, and similar ages for consuming their first drink. Alternatively, these variables may be related to high-intensity binge drinking, but they were not significant in the models here because they shared variance with time thinking about alcohol and depression rating. For example, Table 1 shows that the groups differ in terms of positive urgency when compared directly.

Theories of alcohol use disorder have suggested that problematic use may arise from a variety of altered neural and psychological states. For example, the incentive sensitization theory suggests that repeated alcohol use changes dopaminergic neurons in the midbrain to have increased response to cues for alcohol, leading an individual to crave alcohol (Robinson and Berridge, 1993). In our study, individuals who reported high-intensity binge drinking also reported spending larger amounts of time thinking about alcohol relative to individuals with no high-intensity binge drinking occasions (see Figure 1). In addition to reward, the allostatic model of addiction posits that drug-seeking occurs to offset negative mood states (Koob and Le Moal, 2001). Negative mood manifests neurobiologically as the activation of the extended amygdala and the hypothalamic-pituitary-adrenal axis (Koob and Le Moal, 2001). Although theories have proposed that executive dysfunction underlies alcohol use disorder, we did not find evidence to support this. However, we only used a single variable, positive urgency, to represent executive dysfunction, so it future studies may find that other measures of executive function do relate to high-intensity binge drinking in AUD. High-intensity binge drinking itself may cause the depressed mood and increased incentive salience observed in participants. Prospective studies would be necessary to clarify the direction of this relationship.

This study had several limitations. We used a calendar-based tool for assessing high-intensity binge drinking, which indicates the number of drinks that participants had on each day, but not how the drinks were spaced throughout the day. Thus, we do not know the blood alcohol level or the level of impairment. We analyzed a group of individuals with a diagnosis of an alcohol use disorder and an average age in the late thirties. Thus, the results may not generalize to younger individuals or individuals without an alcohol use disorder. The data analysis was cross-sectional and retrospective, so the models may not predict future binge drinking. Much of our data comes from self-report, so it is possible that participants misreported drinking or other characteristics. In our control group, high-intensity binge drinking was infrequent, so larger sample sizes will be necessary to confirm our findings. Lastly, we used a convenience sample and did not design a study specifically to test our hypotheses, and it is unclear what effect that may have on our results.

In our study, the most important factors associated with high-intensity binge drinking in individuals with AUD were time thinking about alcohol, depression rating, treatment seeking status, and sex. It is therefore possible that treating depressive symptoms and craving will be a helpful way to approach high-intensity binge drinking. Determining the causes and optimal treatment strategies for high-intensity binge drinking is an important goal, as high-intensity binge drinking is a marker of more severe alcohol use disorder and leads to an increased risk of both acute and long-term consequences.

## Data Availability Statement

The datasets used in this study are not publicly available due to ethical concerns regarding patient privacy and original patient consent. The raw data supporting the conclusions of this article will be made available by requests directly to the corresponding authors.

## Ethics Statement

The studies involving human participants were reviewed and approved by National Institutes of Health Institutional Review Board. The patients/participants provided their written informed consent to participate in this study.

## Author Contributions

This manuscript was conceptualized by JG and VR. ND oversaw data collection and protocol management. JG and JM conducted data analysis. JG drafted the manuscript. All authors helped to interpret results and edit the manuscript. All authors approved of the final draft of the manuscript.

## Funding

This study was supported by the NIAAA Division of Intramural Clinical and Biological Research (Z1A AA000466 and Z1A AA000310).

## Conflict of Interest

The authors declare that the research was conducted in the absence of any commercial or financial relationships that could be construed as a potential conflict of interest.

## Publisher’s Note

All claims expressed in this article are solely those of the authors and do not necessarily represent those of their affiliated organizations, or those of the publisher, the editors and the reviewers. Any product that may be evaluated in this article, or claim that may be made by its manufacturer, is not guaranteed or endorsed by the publisher.

## Footnotes

^1^ https://topepo.github.io/caret/index.html
